# Distinct Redox Signalling following Macrophage Activation Influences Profibrotic Activity

**DOI:** 10.1155/2019/1278301

**Published:** 2019-11-11

**Authors:** Caitlin V. Lewis, Antony Vinh, Henry Diep, Chrishan S. Samuel, Grant R. Drummond, Barbara K. Kemp-Harper

**Affiliations:** ^1^Cardiovascular Disease Program, Biomedicine Discovery Institute & Department of Pharmacology, Monash University, VIC 3800, Australia; ^2^Department of Physiology, Anatomy & Microbiology, School of Life Sciences, La Trobe University, VIC 3086, Australia

## Abstract

**Aims:**

To date, the ROS-generating capacities of macrophages in different activation states have not been thoroughly compared. This study is aimed at determining the nature and levels of ROS generated following stimulation with common activators of M1 and M2 macrophages and investigating the potential for this to impact fibrosis.

**Results:**

Human primary and THP-1 macrophages were treated with IFN-*γ*+LPS or IL-4-activating stimuli, and mRNA expression of established M1 (CXCL11, CCR7, IL-1*β*) and M2 (MRC-1, CCL18, CCL22) markers was used to confirm activation. Superoxide generation was assessed by L-012-enhanced chemiluminescence and was increased in both M(IFN-*γ*+LPS) and M(IL-4) macrophages, as compared to unpolarised macrophages (M*Φ*). This signal was attenuated with NOX2 siRNA. Increased expression of the p47phox and p67phox subunits of the NOX2 oxidase complex was evident in M(IFN-*γ*+LPS) and M(IL-4) macrophages, respectively. Amplex Red and DCF fluorescence assays detected increased hydrogen peroxide generation following stimulation with IL-4, but not IFN-*γ*+LPS. Coculture with human aortic adventitial fibroblasts revealed that M(IL-4), but not M(IFN-*γ*+LPS), enhanced fibroblast collagen 1 protein expression. Macrophage pretreatment with the hydrogen peroxide scavenger, PEG-catalase, attenuated this effect.

**Conclusion:**

We show that superoxide generation is not only enhanced with stimuli associated with M1 macrophage activation but also with the M2 stimulus IL-4. Macrophages activated with IL-4 also exhibited enhanced hydrogen peroxide generation which in turn increased aortic fibroblast collagen production. Thus, M2 macrophage-derived ROS is identified as a potentially important contributor to aortic fibrosis.

## 1. Introduction

Macrophages are key cells of the innate immune system and their activation and function is important in tissue homeostasis, disease pathogenesis, and immune regulation [1]. Macrophages exist as a heterogeneous population, continually responding to local microenvironment cues [2]. Over recent years, research has explored the generally opposing roles of two broad populations of macrophage, the “classically activated” proinflammatory M1 macrophage and the “alternatively activated” M2 macrophage. Representing either ends of a spectrum of activation or “polarisation,” M1 and M2 macrophages are thought to play predominant roles in inflammation and tissue repair, respectively, although this paradigm is now considered overly simplified [1–3]. It is now recognized that the precise stimuli for activation are important in determining the characteristics and expression profiles of activated macrophages, which can vary greatly across different *in vitro* and *in vivo* contexts [3]. Nonetheless, understanding how macrophages respond to different cytokines and factors in the microenvironment can inform on their contribution to various disease states and identify new therapeutic targets. To date, the reactive oxygen species (ROS)-generating capacities of macrophages in different activation states have not been thoroughly compared.

The initial infiltration of macrophages to a site of injury leads to the generation of proinflammatory cytokines and ROS. Whilst this defense mechanism contributes to microbial killing, it also exacerbates inflammatory disease. M1 macrophages which are activated by these proinflammatory cytokines (e.g., IFN-*γ*) play a predominant role in this setting, and a contribution of ROS to these processes is evident [4]. Thus, M1 macrophage-derived superoxide, together with inducible nitric oxide synthase (iNOS)-derived nitric oxide (NO), can lead to the generation of the powerful oxidant, peroxynitrite. Whilst peroxynitrite is central to pathogen killing, it can also cause oxidation and nitration of proteins and lipids. Importantly, the ROS-generating capacity of M1 macrophages is reliant predominantly on the activity of the NOX2 isoform of the NADPH oxidase (NOX) family of enzymes which is highly expressed in macrophages. Inflammatory stimuli increase the expression and activity of NOX2 oxidase as an important mechanism of microbial killing [5–7]. Given their well-recognized ROS-generating capacity [8, 9], M1 macrophages have been shown to contribute to inflammation-associated organ damage [10], observed in diseases such as hypertension, diabetes, and kidney disease [11–13].

In the later phases of the disease process, macrophages release anti-inflammatory molecules and growth factors and promote healing and regeneration. Whilst initially beneficial, the healing process becomes pathological when it is continuous, leading to remodeling of the extracellular matrix. Macrophages recruited for these processes, termed “M2” or “alternatively activated” macrophages, are commonly activated by Th2 cytokines, anti-inflammatory cytokines, and growth factors [1]. This is of particular relevance in the setting of hypertension in which we have demonstrated the accumulation of M2 macrophages, as indicated by expression of the marker CD206 (also known as MRC-1), in the vessel wall associated with aortic stiffening and increased collagen deposition [14]. The profibrotic effects of M2 macrophages are generally attributed to their ability to generate transforming growth factor-*β* (TGF-*β*) and platelet-derived growth factor (PDGF) [1, 15], thereby promoting differentiation of fibroblasts to collagen-generating myofibroblasts [16, 17]. Of note, M2 macrophages also express NOX2 oxidase and have the capacity to generate ROS [18]. As such, it is possible that their generation of ROS, particularly hydrogen peroxide, may also contribute to this profibrotic response. Evidence in support of this concept comes from the observation that NOX-derived superoxide can be rapidly converted to hydrogen peroxide, which has been shown to directly stimulate collagen production and myofibroblast differentiation of fibroblasts *in vitro* [19]. Furthermore, the profibrotic effect of coculturing pulmonary fibroblasts with macrophages was shown to be reduced in the presence of the NOX inhibitor apocynin [20]. Thus, ROS may represent a novel mediator of the remodelling actions of M2 macrophages observed in fibrotic diseases, such as lung fibrosis, muscular dystrophy [21, 22] and the aortic stiffening associated with hypertension [14].

To date, it has been generally assumed that M1 macrophages have an enhanced oxidative capacity [2, 8, 9], contributing to their proinflammatory properties and tissue damaging effects. Hence, ROS generation is considered an “M1” function. However, no studies have directly compared the oxidative capacity, NOX2 activity, and the nature of the ROS generated by M1 and M2 macrophages. Whether the amount and type of ROS generated impacts on their functions in disease, particularly with regard to fibrosis, remains to be determined. Inconsistency in the terminology and definition of macrophage subsets and limitations in translation between *in vitro* and *in vivo* macrophages has recently been acknowledged [3]. This study is aimed at elucidating whether macrophages stimulated with commonly used Th1 (IFN-*γ* and LPS) versus Th2 (IL-4) stimuli exhibit a differential capacity to generate ROS. We also aim to compare the nature of the ROS generated and investigate whether this may, in turn, influence their profibrotic capacity. These macrophages will be referred to as M(IFN-*γ*+LPS) and M(IL-4) according to the recommendations from Murray et al. [3].

## 2. Materials and Methods

### 2.1. Primary Human Monocyte Isolation

Primary human monocytes were isolated from healthy blood donor buffy coats (Australian Red Cross Blood Service, Melbourne, Australia). Buffy coats were mixed with phosphate-buffered saline (PBS; without Ca^2+^or Mg^2+^; Sigma-Aldrich) supplemented with 0.5% fetal bovine serum (FBS; Sigma-Aldrich) and 2 mM ethylenediaminetetraacetic acid (EDTA; Sigma-Aldrich) and layered onto Ficoll-Paque PLUS (GE Healthcare no. 17-144) for density gradient centrifugation (400 g, 40 minutes, acceleration = 1, deceleration = 0). The peripheral blood mononuclear cell (PBMC) layer was collected and monocytes isolated using a human pan monocyte isolation kit (Miltenyi Biotec no. 130-096-537), according to the manufacturer's instructions. The purity of the monocyte population was confirmed to be at least 85% as determined by flow cytometry using CD14^+^/CD16^+^ expression.

### 2.2. Monocyte to Macrophage Differentiation and Macrophage Activation

The THP-1 human monocytic cell line was supplied by Dr. Meritxell Canals (Monash Institute of Pharmaceutical Sciences, Parkville, Australia). THP-1 cells were cultured in high-glucose RPMI 1640 medium (Gibco Life Technologies), supplemented with 10% heat-inactivated FBS and grown in T75 tissue culture flasks in a humidified incubator maintained at 37°C with 5% CO_2_ (Sanyo MCO-18AIC CO_2_ incubator, Quantum Scientific). THP-1 monocytes were passaged every 3-4 days and seeded on 6-well plates (1 × 10^6^ cells/well) for RNA and protein extraction or 96-well plates (5 × 10^4^ cells/well) for superoxide and hydrogen peroxide detection. THP-1 monocytes were differentiated to macrophages (M*Φ*) via the addition of 100 nM phorbol-12,13-dibutyrate (PDBu, Calbiochem) for 24 hours. THP-1 macrophages were subsequently left untreated (M*Φ*) or treated with either a combination of 5 ng/ml interferon-*γ* (IFN-*γ*; Sigma-Aldrich no. I3265) and 10 ng/ml lipopolysaccharide (LPS; Sigma-Aldrich no. L2630, E. coli 0111:B4 strain) or with 25 ng/ml interleukin-4 (IL-4; Sigma-Aldrich no. I4269), for 24, 48, or 72 hours for PCR, western blotting, and ROS detection. A subset of macrophages were treated in the presence of 100 nM NOX2 siRNA (Santa-Cruz no. sc-35503) or missense siRNA (control siRNA-A; Santa-Cruz no. sc-37007). These cells were transfected using Lipofectamine RNAiMAX (Gibco Life Technologies) in opti-minimum essential medium (Opti-MEM, Gibco Life Technologies) for 6 hours prior to polarisation in complete RPMI 1640 culture medium.

Isolated donor blood-derived primary monocytes (1 × 10^6^ cells/ml) were differentiated into macrophages by culturing for 7 days in RPMI 1640 GlutaMAX medium (Gibco Life Technologies), supplemented with 10% FBS, 1× antibiotic/antimyotic (Gibco Life Technologies, USA), 1 mM sodium pyruvate (Sigma-Aldrich), 1× nonessential amino acids (NEAA; Gibco Life Technologies), and 50 ng/ml macrophage colony-stimulating factor (M-CSF; Miltenyi Biotec no. 130-096-491). Following a 7-day macrophage differentiation, human primary macrophages were either left untreated (M*Φ*), treated with 100 ng/ml LPS and 20 ng/ml IFN-*γ*, or treated with 25 ng/ml IL-4 in the absence of M-CSF. Cells were treated for either 3, 6, or 24 hours for real-time PCR or for 24 hours for western blotting and L-012-enhanced chemiluminescence.

### 2.3. Aortic Fibroblast Coculture with M(IFN-*γ*+LPS) or M(IL-4) Macrophages

Primary human aortic adventitial fibroblasts (AoAF; Lonza no. CC-7014; Lonza) were grown in Stromal Cell Growth Medium (SCGM; Lonza no. CC-3205), containing 5% FBS and used from passages 2 to 8. Fibroblasts were maintained in T-75 flasks in a humidified incubator at 37°C with 5% CO_2_. Once confluent, AoAFs were passaged in SCGM using Trypsin-EDTA solution (Lonza no. CC-5012) for cell detachment. For coculture experiments, THP-1 macrophages were first stimulated with IFN-*γ*/LPS or IL-4 for 72 hours in complete RPMI 1640 medium in 24-well cell culture inserts (0.4 *μ*m pore, <0.85 × 10^8^ pores/cm^2^, 5 × 10^4^ cells/insert; Thermo Fisher Scientific). The THP-1 medium was replaced with serum-free RPMI 1640 medium, and the inserts were transferred to wells with AoAF (5 × 10^4^ cells/well) in serum-free SCGM. THP-1 macrophages were stimulated with 10 *μ*M PDBu in the presence or absence of 1000 U/ml PEG-catalase (Sigma-Aldrich no. C4963), and the cultures incubated for further 24 hours before lysates were harvested for western blotting.

### 2.4. RNA Extraction and Real-Time PCR

Total RNA was extracted from macrophages using the RNeasy Mini Kit (Qiagen) according to the manufacturer's instructions. RNase-free DNase (Qiagen) was used to remove any contaminating DNA. The amount of RNA in each sample was quantified using the NanoDrop 1000D spectrophotometer (Thermo Scientific), which measures absorbance at 260 nm and 280 nm. An A_260_ : A_280_ ratio of 2 or more was considered sufficiently pure. 1 *μ*g of RNA from each sample was reverse transcribed into cDNA using the High Capacity cDNA Reverse Transcription Kit (Applied Biosystems) with the reaction run in a thermal cycler (Bio-Rad MyCycler, Bio-Rad Laboratories). The resultant cDNA was used as a template for real-time PCR with TaqMan® primers and probes for IL-1*β*, CXCL11, CCR7, MRC-1, CCL22, CCL18, CYBA (p22phox), CYBB (NOX2), NCF1 (p47phox), NCF2 (p67phox), NCF4 (p40phox), NOX1, NOX4, NOX5, SOD1, SOD2, and SOD3 (Applied Biosystems). *β*-Actin and 18S were used as housekeeping genes. Real-time PCR was run in triplicate on the CFX96 Touch™ Real-Time PCR Detection Machine (Bio-Rad Laboratories). Gene expression was normalised to *β*-actin or 18S and quantified relative to the average M*Φ* value using the comparative cycle threshold (Ct) method with the formula: fold change = 2^−ΔΔCt^ [23].

### 2.5. Protein Extraction and Western Blotting

Total protein from macrophage and fibroblast cell lysates was collected in 1.5× Laemmli buffer (7.5% glycerol; 3.75% *β*-mercaptoethanol; 2.25% SDS; 75 mM Tris-HCl pH 6.8; 0.004% bromophenol blue) and 1× RIPA lysis and extraction buffer (Cell Signaling Technology, USA), respectively. Cell debris was cleared by centrifugation (13000 rpm, 10 min, 4°C) and supernatants collected. Protein concentrations were determined using either a modified Lowry protocol (RCDC colorimetric protein assay kit; Bio-Rad Laboratories) or bicinchoninic acid- (BCA-) based colorimetric quantification (Pierce^TM^ BCA Protein Assay, Thermo Scientific). Equivalent volumes of protein in 1.5× Laemmli buffer were loaded into 7.5% or 10% polyacrylamide gels. Proteins were separated by SDS-PAGE and transferred onto low fluorescence polyvinylidene fluoride (LF PVDF) membranes using the Bio-Rad Trans Blot Turbo transfer system (Bio-Rad Laboratories). Membranes were blocked with 5% skim milk in Tris-buffered saline (TBS; 200 mM Tris, 150 mM NaCl, pH 7.5) with 0.1% Tween-20 for 1 hour and subsequently probed with primary antibodies against NOX2 (1 : 500; Santa-Cruz no. sc-130549 (CL5)), p47phox (1 : 1000; BD Transduction Laboratories no. 610354), p67phox (1 : 2000; EMD Millipore no. 07-002), SOD2 (1 : 1000; EMD Millipore no. 06-984), SOD3 (1 : 1000; EMD Millipore no. 07-704), *α*SMA (1 : 2500; Abcam no. ab5694) or collagen 1 (1 : 1000; Abcam no. ab34710), and the housekeeping protein GAPDH (1 : 20000; Abcam no. ab8245) overnight at 4°C. 1 hour incubation with horseradish peroxidase- (HRP-) conjugated anti-rabbit (1 : 10000; Dako) or anti-mouse (1 : 10000; Jackson ImmunoResearch Laboratories) secondary antibody was then performed and protein bands visualised using Clarity ECL substrate (Bio-Rad Laboratories) and the ChemiDoc MP system (Bio-Rad Laboratories). Densitometries of protein bands were quantified using Image Lab Software (Bio-Rad Laboratories) and normalised to the housekeeping protein, GAPDH.

### 2.6. Superoxide Detection via L-012-Enhanced Chemiluminescence

THP-1 or human primary macrophages were seeded and activated on white 96-well tissue culture plates (PerkinElmer) at 5 × 10^4^ and 2 × 10^5^ cells/well, respectively. Groups were set up in quintuplicate with a cell-free control group, comprising media alone, included to provide a background reference. On the day of experimentation, culture medium was removed and cells were washed and incubated in warmed Krebs-HEPES buffer (118 mM NaCl, 4.7 mM KCl, 1.2 mM KH_2_PO_4_, 1.2 mM MgSO_4_·7H_2_O, 2.5 mM CaCl_2_, 25 mM NaHCO_3_, 11.7 mM glucose, 20 mM HEPES; pH 7.4) and background chemiluminescence measured for 30 minutes. Chemiluminescence was measured using a Chameleon Luminescence Plate Reader (Hidex Ltd., Turku, Finland) and data acquired using the MicroWin (Mikrotek, Overath, Germany) data acquisition system. 100 *μ*M L-012 (Wako Pure Chemical Industries) was then added to each well, and basal superoxide levels were monitored for 30 minutes. Finally, the protein kinase C (PKC) activator PDBu (10 *μ*M) was added to each well and superoxide production was then measured for a further 30 minutes. PDBu-stimulated superoxide production was quantified as the average signal over 5 minutes at its peak. The basal signal from each signal was subtracted (average over final 5 minutes). In a subset of experiments, cells were treated with superoxide dismutase (SOD; 1000 U/ml; human recombinant, expressed in *E. coli*; Sigma-Aldrich S9076) just prior to the beginning of the assay, to confirm signal specificity for superoxide.

### 2.7. Hydrogen Peroxide Detection via Amplex Red

THP-1 macrophages were seeded and activated on black 96-well tissue culture plates (PerkinElmer) at 5 × 10^4^ cells/well in quintuplicate. On the day of experimentation, the media was removed, cells rinsed with Krebs-HEPES solution, and Krebs-HEPES added to each well in the absence or presence of 1000 U/ml SOD or PEG-Catalase. Working Amplex Red solution (Invitrogen), comprising of Amplex Red (5 *μ*M) and HRP (0.2 U/*μ*l), was then added to sample wells and fluorescence detected over 90 minutes using a Hidex Chameleon Plate Reader at 37°C (520 nm excitation filter, 590 nm emission filter). Cells were either left unstimulated or stimulated with 10 *μ*M PDBu immediately prior to reading. The final fluorescence measurement from each group was fitted to a hydrogen peroxide standard curve (0-5 *μ*M). Fold changes in hydrogen peroxide concentration were calculated relative to the M*Φ* value recorded on the same day.

### 2.8. Intracellular ROS Detection via DCF

THP-1 macrophages were stimulated with IFN-*γ*/LPS or IL-4 for 72 hours in complete RPMI 1640 medium on 6-well plates at 1 × 10^6^ cells/well. Adherent cells were washed with warmed PBS prior to incubation with a cell permeable ROS-sensitive dye, 5-(and-6)-chloromethyl-2′,7′-dichlorodihydrofluorescein diacetate, acetyl ester (CM-H_2_DCFDA; DCF; 1 *μ*M, Sigma-Aldrich no. D6883) for 30 minutes at 37°C. Cells were then left unstimulated or stimulated with 10 *μ*M PDBu for a further 30 minutes. In a subset of experiments, macrophages were incubated with 1000 U/ml PEG-Catalase for 15 minutes prior to PDBu stimulation. Following stimulation, cells were detached with Accutase® solution (Sigma-Aldrich), centrifuged at 1500 rpm for 5 min and resuspended in PBS. Cells were then analysed on a LSR II flow cytometer (BD Biosciences). Fold changes in DCF fluorescence were calculated relative to the M*Φ* value recorded on the same day.

### 2.9. Statistical Analysis

All data are expressed as mean ± SEM. Comparisons of multiple treatment groups were made using an ordinary or repeated measures one-way analysis of variance (ANOVA) with a Dunnett's or Sidak's post hoc test, respectively. When comparing two groups, a Student's unpaired *t*-test was used. *P* < 0.05 was considered to be statistically significant, and data were graphed and analysed using GraphPad Prism 7.02 software.

## 3. Results

### 3.1. M(IFN-*γ*+LPS) and M(IL-4) Macrophages Express M1 and M2 Markers, Respectively, and Both Phenotypes Demonstrate Enhanced Superoxide Generation

**Figure 1:**
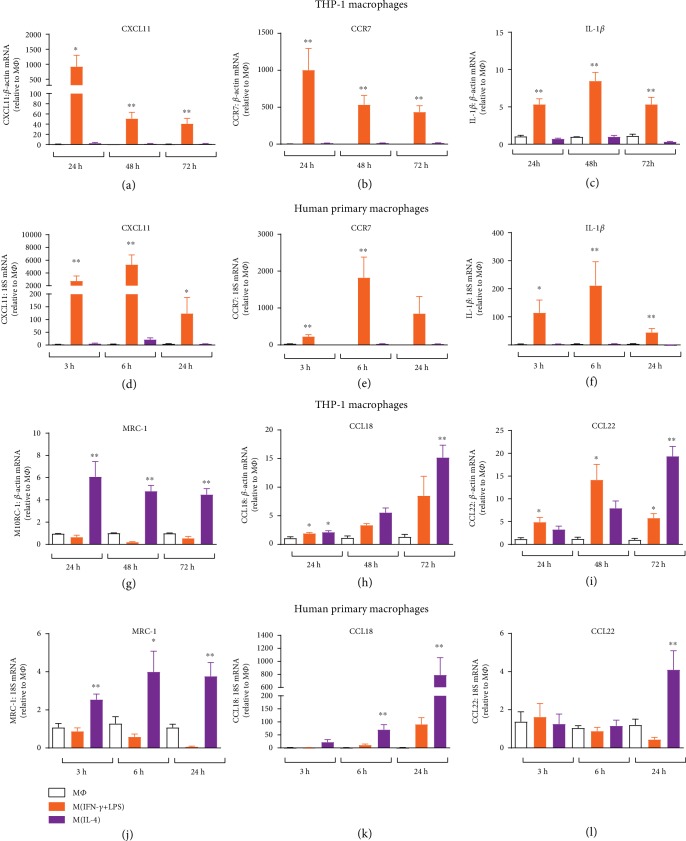
Time course of M1 and M2 marker mRNA expression in human macrophages. PDBu-differentiated THP-1 macrophages or M-CSF-differentiated human primary macrophages were left untreated (M*Φ*) or treated with IFN-*γ* and LPS (THP-1: 5/10 ng/ml; primary: 20/100 ng/ml; IFN-*γ*+LPS) or IL-4 (25 ng/ml) for 3-72 hours. mRNA expression of M1 (CXCL11 (a, d); CCR7 (b, e), and IL-1*β* (c, f)) and M2 (MRC-1 (g, j); CCL18 (h, k); CCL22 (i, l)) markers were determined by RT-qPCR and expressed relative to untreated macrophages (M*Φ*). Results presented as mean ± SEM, *n* = 4‐8. ^∗^*P* < 0.05, ^∗∗^*P* < 0.01 vs. M*Φ* (1-way ANOVA followed by Dunnett's post hoc test).

Treatment of both PDBu-differentiated THP-1 macrophages and M-CSF-differentiated human primary monocyte-derived macrophages, with the combination of IFN-*γ* and LPS, leads to marked increases in the expression of M1 genes (CXCL11, CCR7, and IL-1*β*). In THP-1 cells, CXCL11 (Figure 1(a)) and CCR7 (Figure 1(b)) increased by up to 1000-fold, with the greatest change observed following 24 hours of treatment. IL-1*β* expression increased between 5- and 10-fold throughout the 72-hour treatment period (Figure 1(c)). The magnitude of increase in M1 genes was larger in primary macrophages, with increases of ~5000-fold for CXCL11 (Figure 1(d)), ~2000-fold for CCR7 (Figure 1(e)), and ~200-fold for IL-1*β* apparent at 6 hours (Figure 1(f)). Treatment with IL-4 resulted in an increase in the M2 marker MRC-1 (CD206) by approximately 5-fold throughout the treatment period in both THP-1 and primary macrophages (Figures 1(g) and 1(j)). A time-dependent increase in the M2 markers CCL18 and CCL22 was observed with CCL18 elevated 15- and 800-fold (Figures 1(h) and 1(k)) and CCL22 20- and 5-fold (Figures 1(i) and 1(l)), in THP-1 and primary macrophages, respectively.

**Figure 2:**
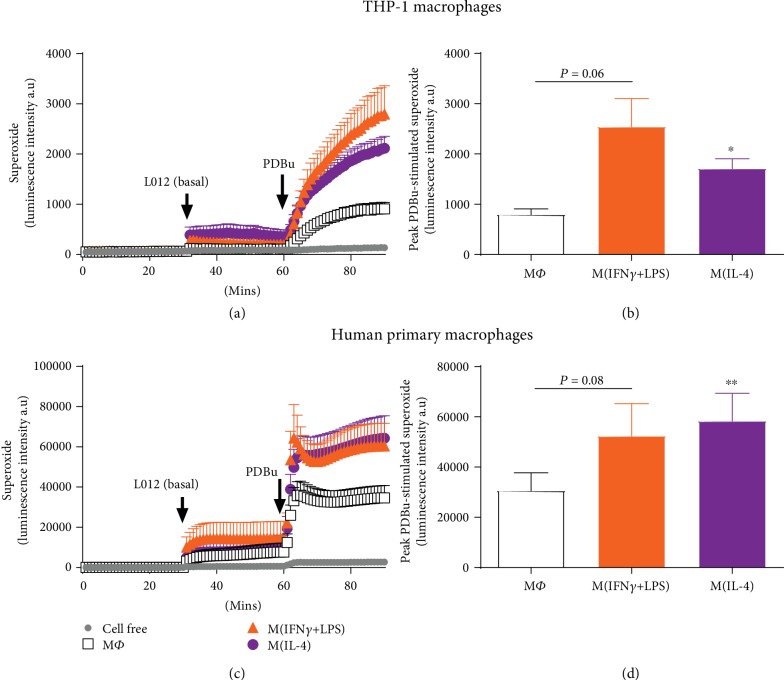
PDBu-stimulated superoxide generation in activated human macrophages. PDBu-differentiated THP-1 macrophages (a, b) or M-CSF-differentiated human primary macrophages (c, d) were left untreated (M*Φ*) or treated with IFN-*γ*+LPS (M1) or IL-4 (M2) for 24 (THP-1) or 72 hours (primary macrophages), and superoxide generation was detected via L-012-enhanced chemiluminescence. Left hand side (LHS): average recordings demonstrating initial background readings (1-30 min), basal superoxide as detected following the addition of L-012 (100 *μ*M; 31-60 min) and PDBu (10 *μ*M)-stimulated superoxide generation (61-90 min) measured as luminescence intensity in arbitrary units (a.u) in (a) THP-1 and (c) primary macrophages. Right hand side (RHS): peak PDBu-stimulated (basal signal subtracted) superoxide generation in (b) THP-1 and (d) primary macrophages. All results presented as mean ± SEM, *n* = 7. ^∗^*P* < 0.05, ^∗∗^*P* < 0.01 vs. M*Φ* (1-way repeated measures ANOVA followed by Dunnett's post hoc test).

Having demonstrated differential activation of macrophages, we next assessed basal and PDBu-stimulated superoxide generation in M(IFN-*γ*+LPS) and M(IL-4) after 72 hours and 24 hours, for THP-1 and human primary macrophages, respectively. In THP-1 cells, whilst basal superoxide levels did not differ significantly between macrophages in different activation states, PDBu-stimulated superoxide generation was increased by ~219% and ~115% in M(IFN-*γ*+LPS) and M(IL-4) macrophages, respectively, as compared to untreated macrophages (Figures 2(a) and 2(b)). Of note, both the basal and PDBu-stimulated superoxide signals were much larger in primary macrophages as compared to THP-1 (Figures 2(c) and 2(d)). Peak superoxide generation in response to PDBu in untreated primary macrophages (30560 ± 7162 counts/sec) was ~30-fold greater than that in untreated THP-1 macrophages (797 ± 112 counts/sec). Nonetheless, PDBu-stimulated superoxide generation was similar in primary M(IFN-*γ*+LPS) and M(IL-4) macrophages and up to 91% greater than that observed in M*Φ* (Figure 2(d)). The L-012 chemiluminescence signal was confirmed to be specific for superoxide via treatment with superoxide dismutase (Supplementary [Supplementary-material supplementary-material-1]).

### 3.2. Differential Regulation of NOX2 Oxidase Subunit Expression following IFN-*γ*+LPS vs. IL-4 Stimulation

**Figure 3:**
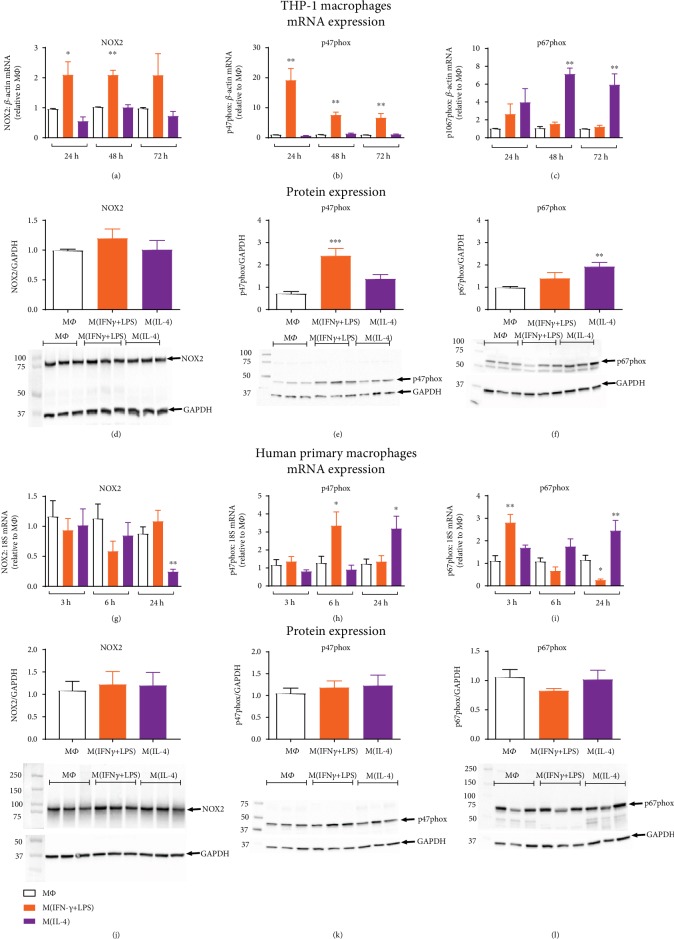
NOX2 oxidase subunit expression in activated human macrophages. PDBu-differentiated THP-1 macrophages or M-CSF-differentiated human primary macrophages were left untreated (M*Φ*) or treated with IFN-*γ*+LPS (M1) or IL-4 (M2) for 3 to 72 hours. Time course of NOX2 (a, g), p47phox (b, h), and p67phox (c, i) mRNA expression determined by RT-qPCR and expressed relative to the average M*Φ* (untreated) value. Protein expression of NOX2 (d, j), p47phox (e, k), and p67phox (f, l) after 24 (primary macrophages) or 72 hours (THP-1 macrophages), determined via western blotting. Representative blots, depicting *n* = 3, are shown below each graph with GAPDH used as a loading control. All results presented as mean ± SEM, *n* = 5‐8. ^∗^*P* < 0.05, ^∗∗^*P* < 0.01, ^∗∗∗^*P* < 0.001 vs. M*Φ* (1-way ANOVA followed by Dunnett's post hoc test).

To examine the mechanisms contributing to increased superoxide generation in both M(IFN-*γ*+LPS) and M(IL-4) macrophages, NOX isoform and subunit expression were assessed. Thus, NOX2 oxidase comprises the membrane-bound catalytic subunits, NOX2 and p22phox, together with the cytosolic regulatory subunits, p47phox, p67phox, and p40phox. In THP-1 macrophages, NOX2 mRNA was increased approximately 2-fold following IFN-*γ*+LPS activation, yet remained unchanged in macrophages activated with IL-4 (Figure 3(a)). Interestingly, mRNA expression of the p47phox subunit was upregulated in M(IFN-*γ*+LPS) macrophages (20-fold at 24 hours; 6-fold at 48 and 72 hours, Figure 3(b)), while the p67phox subunit was upregulated in M(IL-4) macrophages (up to 6-fold at 48 and 72 hours, Figure 3(c)). p22phox and p40phox subunits were unchanged (Supplementary [Supplementary-material supplementary-material-1]). NOX2 protein expression did not appear to differ between macrophages in different activation states (Figure 3(d)); however, the observed changes in p47phox and p67phox mRNA were found to translate to increases in protein expression. p47phox protein was increased approximately 2.5-fold in M(IFN-*γ*+LPS) macrophages (Figure 3(e)) whilst p67phox protein was increased approximately 2-fold in M(IL-4) macrophages (Figure 3(f)). In contrast to THP-1 macrophages, IFN-*γ*+LPS stimulation of human primary macrophages was not associated with a change in NOX2 subunit mRNA expression. Although a decrease in NOX2 mRNA was observed in M(IL-4) macrophages at 24 hours (Figure 3(g)), NOX2 was unchanged at the level of the protein (Figure 3(j)). Whilst a time-dependent increase in p47phox mRNA (6 hours, Figure 3(h)) and p67phox mRNA (24 hours, Figure 3(i)) was also associated with M(IFN-*γ*+LPS) and M(IL-4) stimulation, respectively, in human primary macrophages, such changes were not observed at the level of the protein (Figures 3(k) and 3(l)). Furthermore, no significant differences were observed for p22phox mRNA expression. The p40phox subunit was decreased at the mRNA level in primary M(IFN-*γ*+LPS) macrophages (Supplementary [Supplementary-material supplementary-material-1]), yet whether this translated to a reduction in p40phox protein was not confirmed. NOX1 and NOX4 mRNA could not be detected in either THP-1 or primary macrophages, in any of the activation states (Ct values > 40). NOX5 mRNA expression however was observed in both cell lines and was increased in THP-1 macrophages when activated with IL-4 (Supplementary [Supplementary-material supplementary-material-1]).

**Figure 4:**
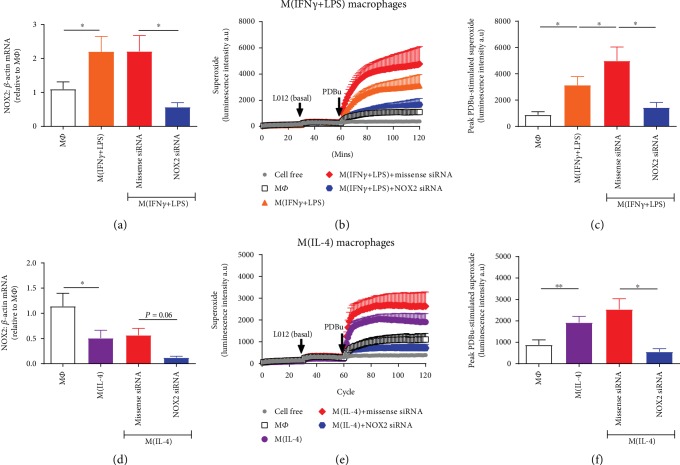
Effect of NOX2 siRNA on M1 and M2 macrophage-derived superoxide. Knockdown of NOX2 mRNA expression in THP-1 macrophages using NOX2 siRNA was confirmed in macrophages treated with both IFN-*γ*+LPS (a) and IL-4 (d) macrophages for 72 hours via RT-qPCR and expressed relative to the average M*Φ* (untreated) value. Effect of NOX2 siRNA and missense siRNA on the PDBu-stimulated superoxide signal in M(IFN-*γ*/LPS) and M(IL-4) macrophages detected via L-012-enhanced chemiluminescence. (b, e) Average recordings demonstrating initial background readings (1-30 min), basal superoxide as detected following the addition of L-012 (100 *μ*M; 31-60 min), and PDBu (10 *μ*M)-stimulated superoxide generation (61-120 min) measured as luminescence intensity in arbitrary units (a.u) in M(IFN-*γ*/LPS) (b) and M(IL-4) (e) macrophages. (c, f) Peak PDBu-stimulated (basal signal subtracted) superoxide generation in M(IFN-*γ*+LPS) (c) and M(IL-4) (f) macrophages. All results presented as mean ± SEM, *n* = 5‐6. ^∗^*P* < 0.05, ^∗∗^*P* < 0.01 vs. M*Φ* (1-way repeated measures ANOVA followed by Dunnett's post hoc test).

To confirm that the superoxide signal in THP-1 macrophages was indeed NOX2 oxidase-derived, NOX2 siRNA was utilised to knock down its expression in all macrophage phenotypes. In M(IFN-*γ*+LPS) and M(IL-4) macrophages, NOX2 mRNA expression was reduced by 74% (Figure 4(a)) and 79% (Figure 4(d)), respectively, following NOX2 siRNA treatment as compared to macrophages treated with missense siRNA. In both phenotypes of macrophages, there was a trend for missense siRNA to increase the PDBu-stimulated superoxide signal to levels above the vehicle-treated cells (Figures 4(c) and 4(f)). Nonetheless, the marked reduction in NOX2 expression in M(IFN-*γ*+LPS) and M(IL-4) macrophages attenuated the superoxide signal to levels similar to those observed in untreated macrophages. Specifically, treatment with NOX2 siRNA reduced the peak PDBu-stimulated superoxide signal in M(IFN-*γ*+LPS) and M(IL-4) by 71% (Figure 4(c)) and 78% (Figure 4(f)), respectively.

### 3.3. M(IL-4) Macrophage Activation Is Associated with Increased Hydrogen Peroxide Generation

**Figure 5:**
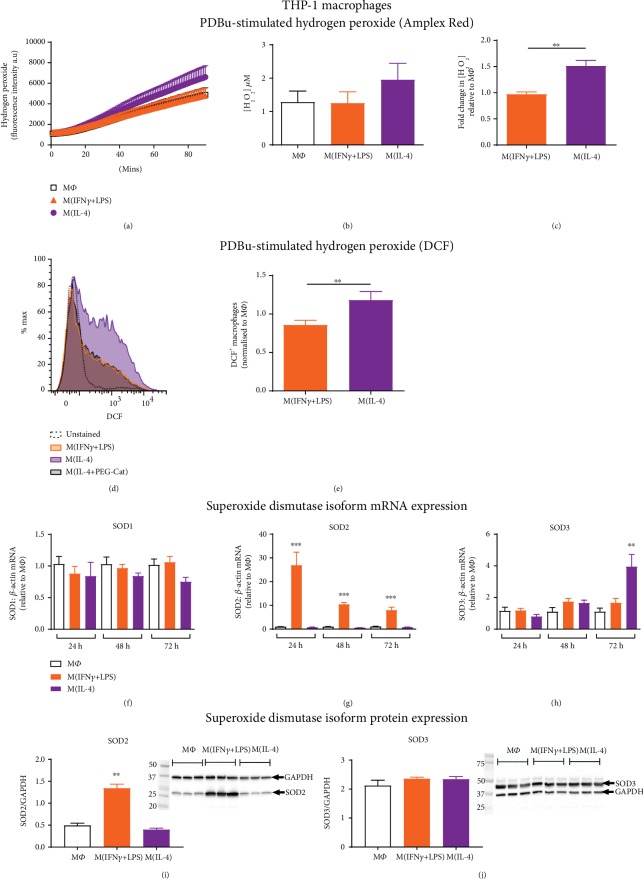
PDBu-stimulated hydrogen peroxide generation in activated human macrophages. PDBu-differentiated THP-1 macrophages were left untreated (M*Φ*) or activated with IFN-*γ*+LPS or IL-4 for 72 hours. (a) Average trace depicting the accumulation of PDBu (10 *μ*M)-stimulated hydrogen peroxide in the culture media over 90 min detected via Amplex Red fluorescence. (b) Hydrogen peroxide concentration was calculated at 90 minutes and expressed as mean concentration ± SEM. (c) M(IFN-*γ*+LPS) and M(IL-4) hydrogen peroxide concentration expressed as a fold change relative to the untreated macrophage (M*Φ*) control, *n* = 8. (d) Representative histogram and gating strategy for DCF^+^ macrophages, depicting M1 versus M2 polarisation and the effect of catalase-polyethylene glycol (PEG-Cat; 1000 U/ml) on the M2 signal. (e) Mean DCF^+^ macrophages, normalised to the response in untreated macrophages (M*Φ*), *n* = 7. (f) SOD1, (g) SOD2, and (h) SOD3 mRNA expression in activated THP-1 macrophages at 24, 48, and 72 hours, determined by RT-qPCR and expressed relative to the average M*Φ* (untreated) value, *n* = 5‐8. (i) SOD2 and (j) SOD3 protein expression in activated THP-1 macrophages at 72 hours, determined by western blotting, *n* = 6. Representative blots, depicting for *n* = 3, shown on RHS, with GAPDH used as a loading control. All results expressed as mean ± SEM, ^∗∗^*P* < 0.01, ^∗∗∗^*P* < 0.001 (1-way ANOVA followed by Dunnett's post hoc test (g, h, i) or Student's unpaired *t*-test (c, e)).

In addition to superoxide, hydrogen peroxide generation was assessed in polarised THP-1 macrophages using two methods, Amplex Red for extracellular and H_2_DCFDA (DCF) for intracellular detection. Of note, a robust basal hydrogen peroxide signal was detected, by both Amplex Red and DCF, in all macrophage phenotypes and was not further modulated by PDBu stimulation (Supplementary [Supplementary-material supplementary-material-1]). Nevertheless, subsequent experiments comparing hydrogen peroxide generation between macrophages in different activation states incorporated PDBu. Amplex Red fluorescence, following PDBu stimulation for 90 minutes, showed a mean hydrogen peroxide concentration of approximately 2 *μ*M for M(IL-4) macrophages, compared to 1 *μ*M in both M(IFN-*γ*+LPS) and M*Φ* (Figure 5(b)). When calculated relative to the M*Φ* signal, there was a 1.5-fold increase in hydrogen peroxide generation following M(IL-4) activation with no change in M(IFN-*γ*+LPS) macrophages (Figure 5(c)). The hydrogen peroxide signal was abolished in the presence of the hydrogen peroxide scavenger, PEG-catalase, and amplified with superoxide dismutase, demonstrating that the assay was specific for hydrogen peroxide (Supplementary [Supplementary-material supplementary-material-1]). To further demonstrate increased hydrogen peroxide production in M(IL-4) macrophages, the intracellular ROS indicator DCF was used and fluorescence detected via flow cytometry (Figures 5(d) and 5(e)). As with the Amplex Red assay, hydrogen peroxide levels were significantly greater in M(IL-4), as compared to M(IFN-*γ*+LPS) macrophages (Figure 5(e)). As shown in the representative histogram (Figure 5(d)), PEG-catalase reduced the signal in M(IL-4) macrophages, suggesting that it was primarily hydrogen peroxide being detected.

To elucidate the potential mechanisms underlying the increased hydrogen peroxide generation following IL-4 activation, we assessed the mRNA expression of different superoxide dismutase isoforms. While SOD1 (cytoplasmic) expression was not altered (Figure 5(f)), SOD2 (mitochondrial) mRNA expression was increased in IFN-*γ*+LPS-activated macrophages by 25-fold at 24 hours and 10-fold at 48 and 72 hours (Figure 5(g)). This change was reflected at the protein level, with a 3-fold increase in SOD2 expression in M(IFN-*γ*+LPS) macrophages at 72 hours (Figure 5(i)). SOD3 (extracellular) mRNA expression was unchanged in response to IFN-*γ*+LPS activation but increased by 4-fold in IL-4-activated macrophages at 72 hours (Figure 5(h)). However, no change in SOD3 protein expression was observed (Figure 5(j)).

### 3.4. Hydrogen Peroxide Generation Contributes to the Profibrotic Activity of M(IL-4) Macrophages

**Figure 6:**
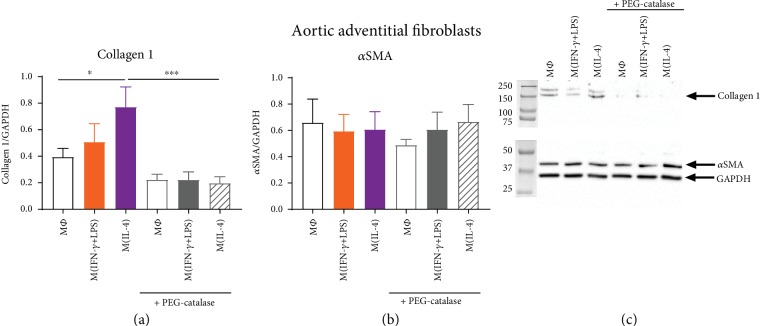
Effect of macrophage coculture on collagen and alpha-smooth muscle actin expression in human aortic adventitial fibroblasts. PDBu-differentiated THP-1 macrophages were left untreated (M*Φ*) or activated with IFN-*γ*+LPS or IL-4 for 72 hours in cell culture inserts. THP-1 macrophages were subsequently transferred to wells containing aortic adventitial fibroblasts, stimulated with 10 *μ*M PDBu in the absence or presence of 1000 U/ml PEG-catalase, and incubated for 24 hours. (a) Collagen 1 and (b) *α*SMA protein were measured in aortic adventitial fibroblasts, *n* = 10‐12. (c) Representative blot, depicting *n* = 1, is shown with GAPDH used as a loading control. Results presented as mean ± SEM, ^∗^*P* < 0.05, ^∗∗∗^*P* < 0.001 (1-way ANOVA followed by Sidak's post hoc test).

To investigate whether M(IL-4) macrophage-derived hydrogen peroxide may contribute to the profibrotic actions of M2 macrophages in the vessel wall, macrophages were cocultured with human aortic adventitial fibroblasts and markers of fibrosis (collagen 1, *α*SMA) measured. Coculture of aortic fibroblasts with M(IL-4) macrophages increased fibroblast collagen 1 expression, as compared to coculture with untreated (M*Φ*) macrophages (Figure 6(a)). PEG-catalase treatment significantly attenuated this effect (Figure 6(a)). Aortic fibroblast *α*SMA expression did not differ in response to coculture with macrophages in distinct activation states (Figure 6(b)), suggesting a lack of effect of macrophage-derived hydrogen peroxide on myofibroblast differentiation.

## 4. Discussion

M1 macrophage infiltration into tissues is a hallmark of inflammatory diseases, and NOX2 oxidase activity has long been associated with M1 function [2]. In this study, we challenge the idea that ROS generation is solely a function of M1 macrophages. Specifically, we demonstrate that activation of macrophages with IL-4 enhances superoxide generation to an equivalent degree as activation of macrophages with IFN-*γ* and LPS and increases hydrogen peroxide production. Of particular relevance to aortic stiffening, an important process involved in the development and clinical consequences of hypertension [24, 25], we reveal a potential role for M2 macrophage-derived hydrogen peroxide in promoting profibrotic responses in aortic adventitial fibroblasts.

Upregulation of NOX2 oxidase activity is observed in macrophages stimulated with the Th1 cytokine IFN-*γ* or bacterial component LPS as part of the response to microbial infection [5, 7]. This has been generally assumed to be a specific M1 macrophage function [15]. Although IL-4 has been shown to inhibit LPS-stimulated, but enhance IFN-*γ*-stimulated, ROS production in macrophages [26], we are the first to demonstrate that it can drive an increase in macrophage superoxide production alone. Importantly, the superoxide signal in both populations of activated macrophages was at least 2-fold higher than in untreated macrophages and was confirmed to be NOX2-derived using NOX2 siRNA. Our findings that stimulation of macrophages with the Th2 cytokine IL-4 results in increased NOX2-derived ROS further support a contribution of NOX2 to the activation of macrophages towards the M2 phenotype, as suggested in previous studies [27, 28]. It should be noted that our findings are not consistent with a study by Kraaij et al. (2013), who reported a reduction in ROS when macrophage differentiation occurred in the presence of IL-4. However, distinct methodological approaches may account for these apparent differences. Thus, Kraaij et al. (2013) treated human primary monocytes with IL-4 during the 7-day M-CSF differentiation period [18], rather than postdifferentiation, as is the more commonly accepted method of M2 macrophage activation [29–31]. As such, this previous study may be indicative of the effects of IL-4 on monocyte to macrophage differentiation rather than on macrophage ROS generation per se.

Although contrary to the generally accepted dogma that M1 macrophages are the major ROS-generating macrophage phenotype [2, 9], our observation that M(IL-4) macrophage function may also involve ROS is perhaps not unexpected. M2 macrophages phagocytose cell debris as part of their tissue repair function [32] and roles for ROS in phagocytosis are well documented [33]. Indeed, ROS have been shown to play a critical signalling role in the activation of macrophages towards the M2 phenotype in both humans and mice [27, 28] and M2 macrophage accumulation and wound healing responses *in vivo* are impaired in the absence of NOX1 and NOX2 in mice [27]. In addition to potential roles in wound healing, a contribution of NOX2-derived ROS to the resolution of inflammation has also emerged [34, 35] and M(IL-10) macrophage-derived ROS have been shown to induce the activation of T-regulatory cells [36]. Collectively, these previous studies support our findings for the involvement of NOX2 oxidase, not only in the function of proinflammatory M1 macrophages but also in other activation states, suggesting roles for NOX2 in immunoregulatory and profibrotic responses in addition to well-known roles in inflammation and microbial killing.

To further investigate the role of NOX2 oxidase in M(IL-4) macrophage ROS generation, we examined the expression of individual NOX2 oxidase subunits. Our findings with regard to NOX2 subunit expression suggest that the mechanism by which each macrophage phenotype increases superoxide differs. Upregulation of the p47phox and, to a lesser extent, the NOX2 catalytic subunit appeared to drive the increase in M(IFN-*γ*+LPS) macrophage superoxide generation. Roles for each of these subunits in promoting NOX2 activity have been shown previously [37, 38]. Furthermore, our results in THP-1 macrophages are consistent with a previous study of human monocytes (MonoMac1 cell line) in which the proinflammatory cytokine, TNF*α*, enhanced p47phox, p67phox, and NOX2 expression with the rank order of expression being p47phox (up to 20-fold) > p67phox (up to 5-fold) > NOX2 (up to 2.5-fold) [37]. NOX2 and p47phox expression are downstream of proinflammatory signalling pathways involving transcription factors associated with M1 macrophage activation such as nuclear factor light chain enhancer of activated B cells (NF*κ*B) [39], signal transducer and activator of transcription (STAT) 1 [40], and interferon regulator factor- (IRF-) 1 [40, 41]. These increases in NOX2 subunit expression in M(IFN-*γ*+LPS) were hence expected. By contrast, IL-4 treatment was associated with upregulation of the p67phox subunit alone. Although yet to be investigated, this may be via the induction of STAT3 [42, 43] and p38 mitogen-activated protein kinase (MAPK) [44–46], signalling pathways utilised by IL-4. It should be acknowledged that a limitation of the current study was that our findings of enhanced p47phox and p67phox expression in M(IFN-*γ*+LPS) and M(IL-4) macrophages were not observed at the protein level in human primary macrophages. This may be due to the heterogeneity of donor blood monocytes as compared to the immortalised THP-1 line, but does question the relevance of these findings and highlights the importance of confirming observations in primary cells.

Collectively, our data support a predominant role of NOX2 oxidase in the generation of superoxide from activated macrophages. However, in agreement with recent reports [47, 48], we also observed NOX5 expression, at least at the mRNA level, in all macrophage phenotypes. Moreover, NOX5 mRNA expression was upregulated by IL-4 in THP-1 cells. Although NOX2 siRNA treatment revealed that the superoxide signal was predominantly NOX2-derived, a contribution of NOX5 to the signal cannot be excluded, particularly considering that it too can be activated via PKC (induced by PDBu stimulation) [49, 50]. Nonetheless, we show for the first time that the superoxide generating capacity of M(IFN-*γ*+LPS) and M(IL-4) macrophages is equivalent and driven predominantly via NOX2 oxidase.

Following generation, superoxide can interact with nitric oxide to form the powerful oxidant, peroxynitrite, or be dismutated to form hydrogen peroxide. Considering the proinflammatory and reparative properties of M1 and M2 macrophages, respectively, we suggest that whilst both phenotypes generate similar levels of superoxide, the final oxidative end product may differ. Importantly, we demonstrated increases in both extracellular and intracellular hydrogen peroxide following activation towards the M2 phenotype with IL-4, an effect which was not evident with M1 activation with IFN-*γ* and LPS. In agreement with this finding for IL-4 stimulation, a previous study reported that augmented macrophage-derived hydrogen peroxide and Cu,Zn-SOD (SOD1) expression were associated with M2 polarisation in the setting of pulmonary fibrosis [51]. Whilst we did not detect a change in SOD1 mRNA, a modest increase in extracellular SOD (SOD3) mRNA was observed following IL-4 treatment, although this did not translate to enhanced SOD3 protein in the macrophage lysates. It should be acknowledged that our investigations of M2 macrophage hydrogen peroxide generation require further exploration to determine the precise mechanisms involved. It remains to be determined if the regulation of catalase or antioxidant enzymes such as glutathionine peroxidase (GPx) and thioredoxin reductase (TrxR) expression may also influence hydrogen peroxide levels in these cells. Furthermore, dismutation of NOX2-derived superoxide may not necessarily be the major source of the hydrogen peroxide signal observed. Given the lack of effect of PDBu stimulation per se on hydrogen peroxide generation, a constitutively active hydrogen peroxide-producing enzyme, such as NOX4, could be involved. Whilst we did not detect NOX4 mRNA in any of our macrophage samples, NOX4 mRNA and protein expressions have been observed in two recent studies of human primary macrophages [52, 53]. Further investigation of NOX4 protein expression and the use of NOX2 and NOX4 siRNA will aid in the identification of the source of M2 macrophage-derived hydrogen peroxide. Future studies should address these limitations in addition to *in vivo* experiments characterising M2 macrophage hydrogen peroxide production in tissues, such as within the vessel wall, during hypertension.

Somewhat surprisingly, M(IFN-*γ*+LPS) macrophages did not demonstrate increased hydrogen peroxide production, despite the increase in superoxide production observed and expression of SOD in these cells. Although not investigated in this study, iNOS is commonly used as a marker of M1 macrophages [2]. Potentially, the iNOS in these cells is out-competing SOD, and thus, the superoxide generated is rapidly reacting with nitric oxide to produce peroxynitrite, rather than being dismutated to form hydrogen peroxide. This remains to be fully investigated. Interestingly, potential for increased mitochondrial hydrogen peroxide generation in M1 macrophages was observed in our study with robust upregulation of the mitochondrial SOD isoform, SOD2, following combined IFN-*γ*/LPS stimulation. This is consistent with findings following TLR receptor activation and LPS stimulation of macrophages [54, 55]. Although increased SOD2 expression was not associated with increased hydrogen peroxide generation in M(IFN-*γ*+LPS) macrophages in our study, we did not specifically investigate mitochondrial ROS, which would not be released from the cell. It remains likely that SOD2 upregulation may be a mechanism by which M1 macrophages are protected against oxidative stress during infection and could be linked with mitochondrial dysfunction, as M1 activation shifts cells from oxidative to glycolytic metabolism [56, 57], compared to M2 activation which is shown to increase mitochondrial content and oxidative phosphorylation [57]. Hence, macrophage phenotype may influence mitochondrial ROS production and mitochondrial dysfunction could contribute to the overall oxidative capacity of M1 macrophages. Intriguingly, increased macrophage thiol oxidative stress, associated with metabolic stress, has been shown to enhance chemotaxis leading to enhanced macrophage recruitment in the setting of atherosclerosis [58]. It would be interesting to investigate whether our activated macrophage cultures also demonstrate alterations in the glutathione (GSH)/glutathione disulphide (GSSG) ratio and chemotactic responses and whether this may also be linked to differences in the bioenergetics of these cells.

Although further investigation of the discussed mechanisms will be important, hydrogen peroxide generation appears to be a function of M(IL-4) macrophages and may be implicated in fibrotic diseases. ROS have been shown to contribute to a multitude of profibrotic and remodelling actions in the vessel wall [59–62]. Thus hydrogen peroxide enhances ROS production in vascular and cardiac cells *in vitro* [19, 63] and promotes remodelling in the intact vasculature [59]. We next sought to determine if M(IL-4) macrophage-derived hydrogen peroxide can promote vascular fibrosis. Indeed, coculture of IL-4-stimulated macrophages with aortic fibroblasts led to increased fibroblast collagen 1 expression, an effect which was negated by the hydrogen peroxide scavenger, PEG-catalase, and was not evident with M(IFN-*γ*+LPS) macrophage coculture. Of note, macrophage-derived hydrogen peroxide, whether it be generated from M*Φ*, M(IFN-*γ*+LPS), or M(IL-4) macrophages, did not appear to contribute to myofibroblast differentiation (detected by *α*SMA expression). Nonetheless, the greater capacity for M(IL-4) macrophages to generate hydrogen peroxide and promote collagen production could contribute to the aortic remodelling and stiffening response observed as a consequence of M2 macrophage accumulation in the vessel wall of hypertensive mice [14]. Future studies should investigate whether a profibrotic effect of M2-derived hydrogen peroxide is also observed in other cells involved in aortic stiffening such as vascular smooth muscle cells and validate the findings in mouse models of hypertension to shed further light on these mechanisms.

## 5. Conclusions

This study compared the ROS-generating capacities of M1 and M2 macrophages and revealed a previously unrecognised role of ROS in M2 macrophage function. Although previously considered a key mediator of M1 macrophage activity, we have shown that increased NOX2-derived superoxide generation also occurs following IL-4-stimulated M2 activation. Furthermore, we show that IL-4 increased macrophage hydrogen peroxide, which promoted enhanced aortic fibroblast collagen synthesis highlighting a potential role in aortic fibrosis. Given the role of M2 macrophages in the development of aortic stiffening during hypertension, our findings may be of particular importance in this setting and we reveal M2-derived ROS as a potential therapeutic target.

## Data Availability

The RT-qPCR, western blot, and ROS detection data used to support the findings of this study are available from the corresponding author upon request.

## Supplementary Materials

Supplementary Materials
*Superoxide and hydrogen peroxide generation in M(IFN-γ+LPS) and M(IL-4) macrophages*. Basal and PDBu-stimulated superoxide generation in M(IFN-*γ*+LPS) and M(IL-4) after 72 hours and 24 hours, for THP-1 and human primary macrophages, respectively, was assessed using L-012 chemiluminescence. The L-012 chemiluminescence signal was confirmed to be specific for superoxide via treatment with superoxide dismutase (Supplementary Figure 1a-d). In addition to superoxide, hydrogen peroxide generation was assessed in polarised THP-1 macrophages using two methods, Amplex Red for extracellular and H_2_DCFDA (DCF) for intracellular detection. Of note, a robust basal hydrogen peroxide signal was detected, by both Amplex Red and DCF, in all macrophage phenotypes and was not further modulated by PDBu stimulation (Supplementary Figure 1e and f). The hydrogen peroxide signal was abolished in the presence of the hydrogen peroxide scavenger, PEG-catalase, and amplified with superoxide dismutase, demonstrating that the assay was specific for hydrogen peroxide (Supplementary Figure 1 g and h). *Differential regulation of NOX2 oxidase subunit expression following IFN-γ+LPS vs. IL-4 stimulation*. To examine the mechanisms contributing to increased superoxide generation in both M(IFN-*γ*+LPS) and M(IL-4) macrophages, NOX isoform and subunit expression were assessed. Thus, NOX2 oxidase comprises the membrane-bound catalytic subunits, NOX2 and p22phox, together with the cytosolic regulatory subunits, p47phox, p67phox, and p40phox. No significant differences were observed for p22phox mRNA expression in THP-1 and primary M(IFN-*γ*+LPS) and M(IL-4) macrophages (Supplementary Figure 2a and d). The p40phox subunit was decreased at the mRNA level in primary M(IFN-*γ*+LPS) macrophages (Supplementary Figure 2d and e), yet whether this translated to a reduction in p40phox protein was not confirmed. NOX1 and NOX4 mRNA could not be detected in either THP-1 or primary macrophages, in any of the activation states (Ct values > 40). NOX5 mRNA expression however was observed in both cell lines and was increased in THP-1 macrophages when activated with IL-4 (Supplementary Figure 2c and f).
