# Supplementary material for: Distinct Redox Signalling following Macrophage Activation Influences Profibrotic Activity
Source: J Immunol Res. 2019 Nov 11;2019:1278301. doi: 10.1155/2019/1278301 (PMC6877990; doi:10.1155/2019/1278301)
Supplement: Supplementary Materials — Superoxide and hydrogen peroxide generation in M(IFN-γ+LPS) and M(IL-4) macrophages. Basal and PDBu-stimulated superoxide generation in M(IFN-γ+LPS) and M(IL-4) after 72 hours and 24 hours, for THP-1 and human primary macrophages, respectively, was assessed using L-012 chemiluminescence. The L-012 chemiluminescence signal was confirmed to be specific for superoxide via treatment with superoxide dismutase (Supplementary Figure 1a-d). In addition to superoxide, hydrogen peroxide generation was assessed in polarised THP-1 macrophages using two methods, Amplex Red for extracellular and H2DCFDA (DCF) for intracellular detection. Of note, a robust basal hydrogen peroxide signal was detected, by both Amplex Red and DCF, in all macrophage phenotypes and was not further modulated by PDBu stimulation (Supplementary Figure 1e and f). The hydrogen peroxide signal was abolished in the presence of the hydrogen peroxide scavenger, PEG-catalase, and amplified with superoxide dismutase, demonstrating that the assay was specific for hydrogen peroxide (Supplementary Figure 1 g and h). Differential regulation of NOX2 oxidase subunit expression following IFN-γ+LPS vs. IL-4 stimulation. To examine the mechanisms contributing to increased superoxide generation in both M(IFN-γ+LPS) and M(IL-4) macrophages, NOX isoform and subunit expression were assessed. Thus, NOX2 oxidase comprises the membrane-bound catalytic subunits, NOX2 and p22phox, together with the cytosolic regulatory subunits, p47phox, p67phox, and p40phox. No significant differences were observed for p22phox mRNA expression in THP-1 and primary M(IFN-γ+LPS) and M(IL-4) macrophages (Supplementary Figure 2a and d). The p40phox subunit was decreased at the mRNA level in primary M(IFN-γ+LPS) macrophages (Supplementary Figure 2d and e), yet whether this translated to a reduction in p40phox protein was not confirmed. NOX1 and NOX4 mRNA could not be detected in either THP-1 or primary macrophages, in any of the activation states (Ct v [file 1278301.f1.pdf]

The following supplementary figures accompany the manuscript

Distinct redox signalling following macrophage activation influences pro-fibrotic activity

Caitlin V. Lewis, Antony Vinh, Henry Diep, Chrishan S Samuel, Grant R. Drummond, Barbara K. Kemp-Harper

Results

Superoxide and hydrogen peroxide generation in M(IFN-γ + LPS) and M(IL-4) macrophages

Basal and PDBu-stimulated superoxide generation in M(IFN-γ + LPS) and M(IL-4) after 72 hours and 24 hours, for THP-1 and human primary macrophages, respectively was assessed using L-012 chemiluminescence. The L-012 chemiluminescence signal was confirmed to be specific for superoxide via treatment with superoxide dismutase (Supplementary Figure 1a-d).

In addition to superoxide, hydrogen peroxide generation was assessed in polarised THP-1 macrophages using two methods, Amplex Red for extracellular, and H_2DCFDA (DCF) for intracellular detection. Of note, a robust basal hydrogen peroxide signal was detected, by both Amplex Red and DCF, in all macrophage phenotypes and was not further modulated by PDBu stimulation (Supplementary Figure 1e and f). The hydrogen peroxide signal was abolished in the presence of the hydrogen peroxide scavenger, PEG-catalase and amplified with superoxide dismutase, demonstrating that the assay was specific for hydrogen peroxide (Supplementary Figure 1g and h).

Differential regulation of NOX2 oxidase subunit expression following IFN-γ + LPS vs IL-4 stimulation

To examine the mechanisms contributing to increased superoxide generation in both M(IFN-γ + LPS) and M(IL-4) macrophages, NOX isoform and subunit expression were assessed. Thus NOX2 oxidase comprises the membrane-bound catalytic subunits, NOX2 and p22phox, together with the cytosolic regulatory subunits, p47phox, p67phox and p40phox. No significant differences were observed for p22phox mRNA expression in THP-1 and primary M(IFN-γ + LPS) and M(IL-4) macrophages (Supplementary Figure 2a and d). The p40phox subunit was decreased at the mRNA level in primary M(IFN-γ + LPS) macrophages (Supplementary Figure 2d and e), yet whether this translated to a reduction in p40phox protein was not confirmed. NOX1 and NOX4 mRNA could not be detected in either THP-1 or primary macrophages, in any of the activation states (Ct values > 40). NOX5 mRNA expression however was observed in both cell lines and was increased in THP-1 macrophages when activated with IL-4 (Supplementary Figure 2c and f).

Supplementary Figure 1

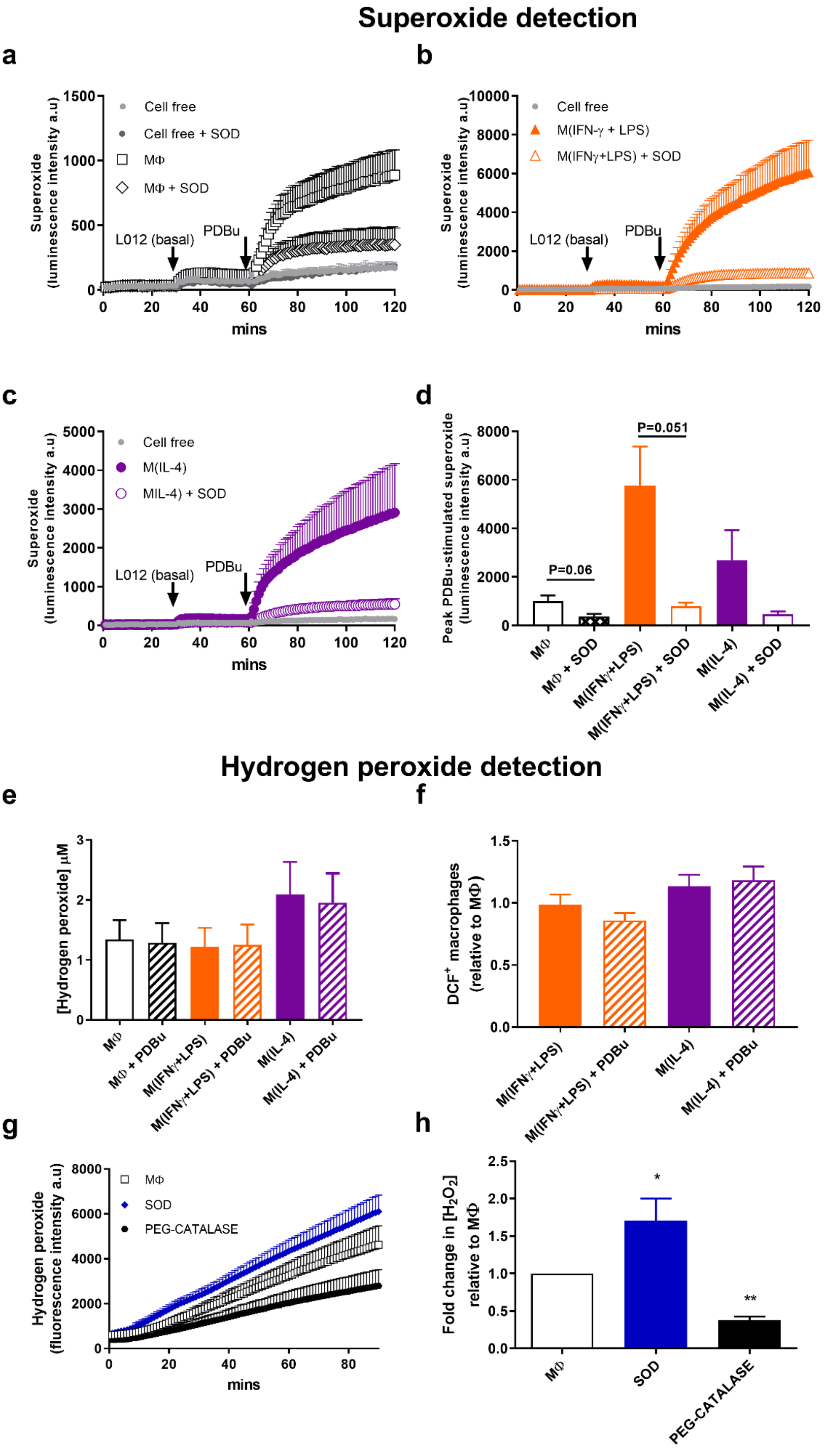

Supplementary Figure 1. Confirmation of superoxide and hydrogen peroxide detection in L-012-enhanced chemilumiscence and Amplex Red assays and effect of PDBu stimulation on hydrogen peroxide signals. a-c) Average recording demonstrating initial background readings (1-30 minutes), basal superoxide as detected following the addition of L-012 (100µM; 31-60 minutes) and PDBu (10 µM)-stimulated superoxide generation (61-120 minutes) measured as luminescence intensity in arbitrary units (a.u). The effect of superoxide dismutase (SOD; 1000 U/ml) on the signal in response to PDBu is shown in unpolarised (MΦ; a), IFN$_{Y}$+LPS treated (M1) (b) and IL-4 treated (M2) (c) macrophages. d) Quantification of the peak chemiluminescent signal in response to PDBu revealed that SOD diminished the response, confirming superoxide detection in this assay, mean ± SEM n=4. e-h) Hydrogen peroxide signals in polarised THP-1 macrophages either left untreated or stimulated with 10 µM PDBu as detected with Amplex Red fluorescence (E; 90 minutes) or f) DCF fluorescence, normalised to the number of DCF^{+} macrophages in the untreated (MΦ) group, mean ± SEM, n=7. g) Average trace depicting the accumulation of PDBu (10 µM)-stimulated hydrogen peroxide in the culture media over 90 mins detected via Amplex Red fluorescence in unpolarised macrophages (MΦ) in the absence or presence of either 1000 U/ml SOD or PEG-catalase. h) Hydrogen peroxide concentration was calculated at 90 minutes and expressed relative to the untreated macrophages (MΦ) control on the day of assay, mean ± SEM. SOD enhances, whilst PEG-catalase diminishes, the signal, confirming hydrogen peroxide detection in this assay, mean ± SEM, n=3-5, *P<0.05, **P<0.01 vs MΦ (1-way repeated measures ANOVA followed by Sidak's post hoc test).

Supplementary Figure 2

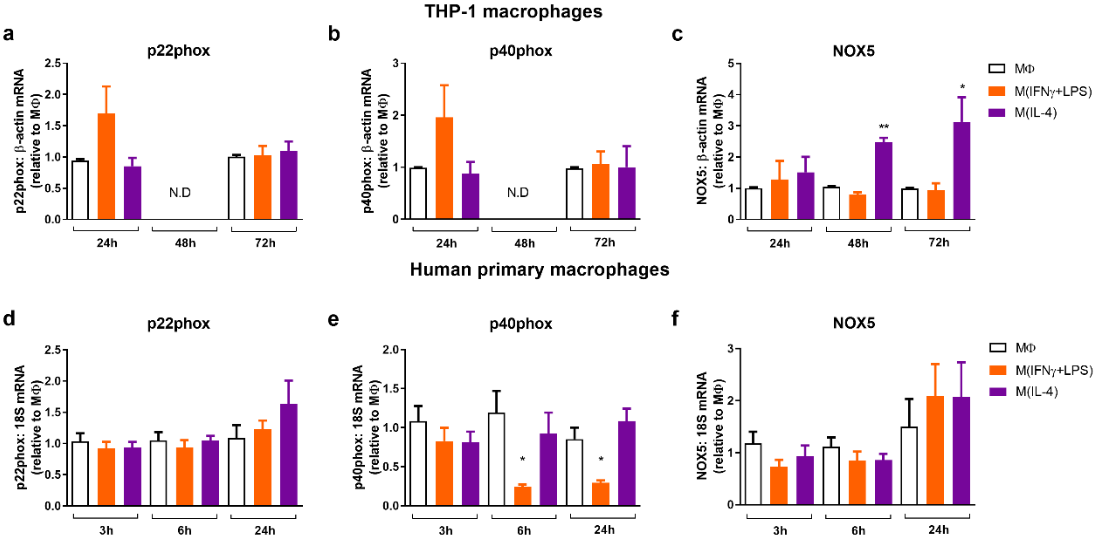

Supplementary Figure 2. Expression of additional NOX2 oxidase subunits and NOX5 in polarised THP-1 and human primary macrophages. PDBu-differentiated THP-1 macrophages (a-c) or M-CSF-differentiated human primary macrophages (d-f) were left untreated (MΦ) or treated with IFN-γ+LPS (5 or 20 ng/ml IFN-γ + 10 or 100 ng/ml LPS) or IL-4 (25 ng/ml) for 3-72 hours. mRNA expression of p22phox (a and d), p40phox (b and e) and NOX5 (c and f) were determined by RT-PCR and expressed relative to the average MΦ (untreated) value. NOX1 and NOX4 isoforms were not detected in any treatment group in either cell line (Ct > 40). Results presented as mean ± SEM, n=4-8. *$P<0.05$, **$P<0.01$ vs MΦ (1-way ANOVA followed by Dunnett's post hoc test). N.D = not determined.
